# LIF–STAT signaling in decidual cells: a possible role in embryo implantation and early pregnancy

**DOI:** 10.1530/JME-24-0006

**Published:** 2024-05-31

**Authors:** Hsien-Ming Wu, Liang-Hsuan Chen, Wei-Jung Chiu, Chia-Lung Tsai

**Affiliations:** 1 Department of Obstetrics and Gynecology, Chang Gung Memorial Hospital Linkou Medical Center, Chang Gung University School of Medicine, Taoyuan, Taiwan

**Keywords:** extracellular vesicle, decidua, microRNA, endometrium, LIF, STAT

## Abstract

In this study, we investigate the effects of miRNA-138-5p and probable G-protein coupled receptor 124 (GPR124)-regulated inflammasome and downstream leukemia inhibitory factor (LIF)–STAT and adhesion molecule signaling in human decidual stromal cells. After informed consent was obtained from women aged 25–38 years undergoing surgical termination of the normal pregnancy and spontaneous miscarriage after 6–9 weeks of gestation, human decidual stromal cells were extracted from the decidual tissue. Extracellular vesicles (EVs) with microRNA (miRNA) between cells have been regarded as critical factors for embryo–maternal interactions on embryo implantation and programming of human pregnancy. MicroRNA-138-5p acts as the transcriptional regulator of GPR124 and the mediator of downstream inflammasome. LIF-regulated STAT activation and expression of integrins might influence embryo implantation. Hence, a better understanding of LIF–STAT and adhesion molecule signaling would elucidate the mechanism of microRNA-138-5p- and GPR124-regulated inflammasome activation on embryo implantation and pregnancy. Our results show that microRNA-138-5p, purified from the EVs of decidual stromal cells, inhibits the expression of GPR124 and the inflammasome, and activates the expression of LIF–STAT and adhesion molecules in human decidual stromal cells. Additionally, the knockdown of GPR124 and NLRP3 through siRNA increases the expression of LIF–STAT and adhesion molecules. The findings of this study help us gain a better understanding the role of EVs, microRNA-138-5p, GPR124, inflammasomes, LIF–STAT, and adhesion molecules in embryo implantation and programming of human pregnancy.

## Introduction

The functional cross talk between embryos and maternal endometrium has a critical role in successful embryo implantation, placentation, and subsequent pregnancy (Gellersen *et al.* 2010, Teklenburg *et al.* 2010, Wu *et al.* 2015). Due to the rejection of the maternal immune system in the process of embryo implantation (Blois *et al.* 2007), the specific embryo–maternal interactions through active intracellular and released protein changes in the peri-implantation phase are shown to promote successful formation of pregnancy (Greening *et al.* 2016). The role of extracellular vesicles (EVs) and microRNAs (miRNA) in decidualization for embryo–maternal interactions of decidual stromal cells has now been investigated in the process of embryo implantation (Wu *et al.* 2022). miRNAs may contribute to inflammation and vascular alteration associated with other mechanisms, such as embryonal development or the tissue remodeling and integrity of the angiogenesis, still needs to be investigated. Leukemia inhibitory factor (LIF) is one growth factor that controls multiple biological functions. During the process of embryo implantation, LIF reveals its expression and actions on the embryos and endometrium (Cullinan *et al.* 1996, Fitzgerald *et al.* 2008). Under *in vitro* conditions, LIF might modulate embryo–endometrial interactions by activating STAT3 and integrins to enhance embryo implantation (Ceydeli *et al.* 2006, Suman *et al.* 2013). Therefore, an extensive investigation of LIF–STAT and integrin signaling may improve the outcome of embryo implantation and subsequent pregnancy.

In this study, we have tried to identify extracted EVs and miRNAs from decidua and decidual stromal cells. miRNA-related inflammasomes and subsequent mechanisms of angiogenesis and tissue remodeling are still not well known. Our study aimed to identify new EV-related miRNAs involved in embryo implantation and pregnancy. We investigated the downregulation of miR-138-5p through the target gene of miR-138a-5p and downstream signaling. The NLRP3 inflammasome is considered important in immunity and human pathophysiology, but the mechanism of microRNA-138-5p-regulated NLRP3 inflammasome and downstream signaling are yet to be elucidated. In the current study, we investigate the roles of miRNA-138-5p, probable G-protein coupled receptor 124 (GPR124), NLRP3 inflammasome, and subsequent LIF–STAT and integrin signaling in embryo implantation and early pregnancy.

## Materials and methods

### Cell culture

To explore the effects of miRNA-138-5p and GPR124-regulated inflammasome and downstream LIF–STAT signaling in human decidual stromal cells, after obtaining informed consent from women aged 25–38 years undergoing surgical termination of normal pregnancy and spontaneous miscarriage after 6–9 weeks of gestation, human decidual stromal cells were extracted from the decidual tissue. The involvement of human subjects in this study was approved by the Institutional Review Board of Chang Gung Memorial Hospital (CGMH-IRB nos. 201601676A3, 201702112B0, 201802242A3, 201902015B0, 202002376B0, and 202100385B0). Enzymatic digestion and mechanical dissociation were applied to purify human decidual stromal cells from the decidual tissue according to a modified protocol (Chou *et al.* 2003). Briefly, the human decidual tissue was minced and treated with type IV collagenase (Sigma-Aldrich) and DNase type I in a shaking water bath at 37°C for 90 min. The cell digest was then passed through a 70 μm filter. The decidual epithelial and stromal cells were collected in a 50 mL tube. Next, decidual epithelial cells were separated from decidual stromal with a 45 μm filter. The decidual stromal cells were subsequently pelleted by centrifugation at 1000 ***g*** for 5 min at room temperature. The cell pellet was washed once, resuspended, and plated in Dulbecco’s modified Eagle’s medium (DMEM) containing 25 mM glucose, l-glutamine, antibiotics (100 U/mL penicillin and 100 μg/mL streptomycin), and supplemented with 10% fetal bovine serum (FBS).

### Extracellular vesicle isolation

For isolation of EVs, the primary decidual stromal cells were cultured in DMEM, including 25 mM glucose, 200 mM l-glutamine, and antibiotics (100 U/mL penicillin and 100 μg/mL streptomycin), and supplemented with 10% FBS at 37°C, 5% CO_2_, following which the medium was converted to DMEM supplemented with Gibco™ extracellular vesicle-depleted FBS (Thermo Fisher, #A2720801) for 72 hours, and the supernatants were collected for isolation of EVs. Then, the culture medium was centrifuged at 1200 ***g*** for 10 min to remove impure cells and filtration through 0.22 μm filters to remove cell debris and particles larger than 200 nm, then centrifuged twice at 10,000 ***g*** for 30 min to remove larger microvesicles (MVs). EVs were pelleted by ultracentrifugation at 100,000 ***g*** for 60 min (Optima XE-90, Beckman Coulter, rotor Ti SW28) (all steps were performed at 4°C). The EVs were dissolved in 100 μL of PBS solution to prepare a suspension, which was preserved at −80°C.

### Extracellular vesicle RNA isolation

TRI Reagent LS (750 μL) (Sigma, #T3934) was added to 250 µL EV sample. Subsequently, 200 µL of chloroform were added to each sample, mixed thoroughly by shaking for over 30 s, and incubated at room temperature for 10 min. Phase separation was performed by centrifugation at 12,000 ***g*** at 4°C for 15 min. The upper aqueous phase was collected. A volume of 1 µL glycogen solution (20 μg/μL) and an equal volume of isopropanol were added to each sample for RNA precipitation. Samples were mixed, incubated 1.5 h at −80°C, and then centrifuged at 12,000 ***g*** at 4°C for 30 min to pellet RNAs. The pellet was washed with 75% ethanol and centrifuged at 12,000 ***g*** at 4°C for 10 min. Pellets were dried for 10 min before resuspending in 15 μL diethyl pyrocarbonate (DEPC)-treated water, and purified RNA was quantified using NanoDrop. Total extracellular vesicle RNAs were reverse transcribed to cDNAs using TaqMan™ MicroRNA Reverse Transcription Kit according to the manufacturer’s instructions. Quantitative PCR was performed using TaqMan™ MicroRNA assay in Applied Biosystems™ QuantStudio™ 5 Real-Time PCR System. All TaqMan™ MicroRNA assays (hsa-miR-138-5P: 0022084; U6 snRNA: 001973) were purchased from Applied Biosystems. U6 snRNA served as the internal control for miRNAs. Quantitative PCR conditions were: pre-denaturation at 95°C, 10 min followed by 95°C 15 s, 60°C 60 s, and totally 40 cycles to detect the expression of miR-138-5P and U6 snRNA. Expression levels are triplicated as the cycle threshold (Ct) value of the candidate gene relative to the Ct value of the housekeeping gene. Fold changes in the expression of each miRNA were calculated by a comparative threshold cycle (Ct) method using the formulas:

ΔCt = Ct_miRNA_ − Ct_U6_

ΔΔCt = ΔCt_case extracellular vesicle-miRNA-ΔCt normal human exosome-miRNA_

### Nanoparticle tracking analysis

The isolated EVs were evaluated using NanoSight NS300 (Malvern Instruments, Worcestershire, UK) equipped with a blue laser (488 nm). The laser illuminated the nanoparticles, and their Brownian motion was captured for 60 s. The video was subjected to nanoparticle tracking analysis (NTA) using the NanoSight particle tracking software to calculate nanoparticle concentrations and size distribution.

### Extracellular vesicle visualization by transmission electron microscopy

The sample was fixed with 2.5% glutaraldehyde for 30 min at room temperature. Extracellular vesicle suspension of 10–20 μL was absorbed onto formvar carbon-coated copper electron microscopy grids (300 mesh) at room temperature for 5 min and then subjected to 3% uranyl acetate staining for 30 min. Grids were washed three times with PBS and were maintained in a semi-dry state before observation by TEM (JEOL JEM-1400, Tokyo, Japan).

### Reverse transcription quantitative polymerase chain reaction

Total extracellular vesicle RNAs were reverse transcribed to cDNAs using TaqMan™ MicroRNA Reverse Transcription Kit (Applied Biosystems PN: 4366596) with primers for miR-138-5p and U6 small nuclear RNA (hsa-miR-138-5p (assay ID: 002284); U6 snRNA (assay ID: 001973), Applied Biosystems) according to the manufacturer’s instructions. Quantitative PCR was performed using QuantStudio™ 5 Real-Time PCR System. Real-time PCR reactions with cDNAs were performed in a 20 μL final volume. A reaction mix containing 2 μL cDNAs, 10 μL 2 × TaqMan® Universal PCR MasterMix II, no UNG (#4440040) and 1 μL 1 × TaqMan™ MicroRNA assay (20×; #4427975), and 7 μL DEPC-treated water was loaded into each well. All of TaqMan™ MicroRNA assays (hsa-miR-138-5p (assay ID: 002284); U6 snRNA (assay ID: 001973), Applied Biosystems) were purchased from Life Technologies. U6 snRNA served as the internal control for miRNAs. Quantitative PCR conditions were: pre-denaturation at 95°C, 10 min followed by 40 cycles of 95°C for 15 s, and 60°C for 1 min to detect the expression of miR-138-5P and U6 snRNA. Expression levels are triplicated as cycle threshold (Ct) value of the candidate gene relative to the Ct value of the housekeeping gene. Fold changes in expression of each miRNA were calculated by a comparative threshold cycle (Ct) method using the formulas:

ΔCt = Ct_miRNA_ − Ct_U6_

ΔΔCt = ΔCt_case exosome-miRNA_ − ΔCt_normal human exosome-miRNA_

### Immunoblot analysis

The cells were lysed in a buffer containing 20 mM Tris, pH 7.4, 2 mM EGTA, 2 mM Na_2_VO_3_, 2 mM Na_4_P_2_O_7_, 2% Triton X-100, 2% SDS, 1 μM aprotinin, 1 μM leupeptin, and 1 mM PMSF. The protein concentration was measured with a protein assay kit using BSA standards according to the manufacturer’s instructions (Bio-Rad Laboratories). Equal amounts of cell lysate were separated by SDS-PAGE and transferred to a nitrocellulose membrane (Hybond-C, Amersham Pharmacia Biotech Inc.). Following blocking with Tris-buffered saline containing 5% non-fat dry milk for 1 h, the membranes were incubated overnight at 4°C with anti-TSG101 (Cell Signaling), anti-CD63 (Cell Signaling), anti-CD9 (Cell Signaling), anti-CD81 (Cell Signaling), anti-GPR124 (Cell Signaling), anti-NLRP3 (Cell Signaling), anti-interleukin (IL-18) (Cell Signaling), anti-IL-1β (Cell Signaling), anti-ASC (Cell Signaling), anti-LIF (Cell Signaling), anti-p-STAT3 (Cell Signaling), anti-STAT3 (Cell Signaling), or anti-integrin β (Cell Signaling) antibody followed by incubation with HRP-conjugated secondary antibody. The immunoreactive bands were detected with an enhanced chemiluminescence kit. The membrane was then stripped with stripping buffer (62.5 mM Tris, 10 mM DTT, and 2% SDS, pH 6.7) at 50°C for 30 min and re-probed with anti-β-actin and anti-α-tubulin antibody (Santa Cruz) as a loading control.

### Small interfering RNA transfection

SiCONTROL NON-TARGETING pool siRNA, siGENOME ON-TARGETplus SMARTpool human GPR124 siRNA, and NLRP3 siRNA were purchased from Dharmacon. The cells were transfected with siRNA (50 nM) using Lipofectamine RNAiMAX. After a 48 h transfection, the medium was removed and changed to fresh serum-free medium. To examine the siRNA transfection, cells were transfected with 50 nM si-GLO (Dharmacon) for 48 h. The transfection efficiency was examined by fluorescent microscopy.

### Enzyme-linked immunosorbent assay

Human primary decidual stromal cells were transfected with si-NLRP3 (50 nM), si-GPR124 (50 nM), or miR-138-5P (25 nM) for 48 h. The endometrial stromal cell medium was collected at 48 h after transfection to assess GPR124, NLRP3, IL-1β, IL-18, ASC, LIF, pSTAT3, STAT3, and intergrin β levels. The protein levels of human IL-1β (EZ-Set^TM^ ELISA Kit: EZ0392) and IL-18 (PicoKine^TM^ ELISA: EK0864) were measured in the endometrial stromal cell supernatants using ELISA kits from BOSTER ELISA kit. Human GPR124, ASC, LIF, pSTAT3, STAT3, intergrin β, and NLRP3 (ab274401) were detected using Abcam ELISA kits. ELISAs were performed following the manufacturer’s instructions.

### Protein quantification assays

Cell culture supernatants were analyzed, at the prescribed times, for the existence of protein activity by using mouse GPR124, NLRP3, ASC, LIF, IL-1β, IL-18, pSTAT3, STAT3, and intergrin β DuoSet ELISA (R&D and MBL) following the manufacturer’s instructions. ATP was quantified in cell supernatants using ATP Determination Kit (Life Technologies).

### Statistical analysis

The results are expressed as the means ± s.e.m. Statistical evaluation was executed with the *t*-test for paired data. Multiple comparisons were first analyzed by one-way ANOVA, followed by Tukey’s multiple comparison tests. A *P*-value of <0.05 was considered statistically significant.

## Results

### Extraction and characterization of extracellular vesicles

**Figure 1:**
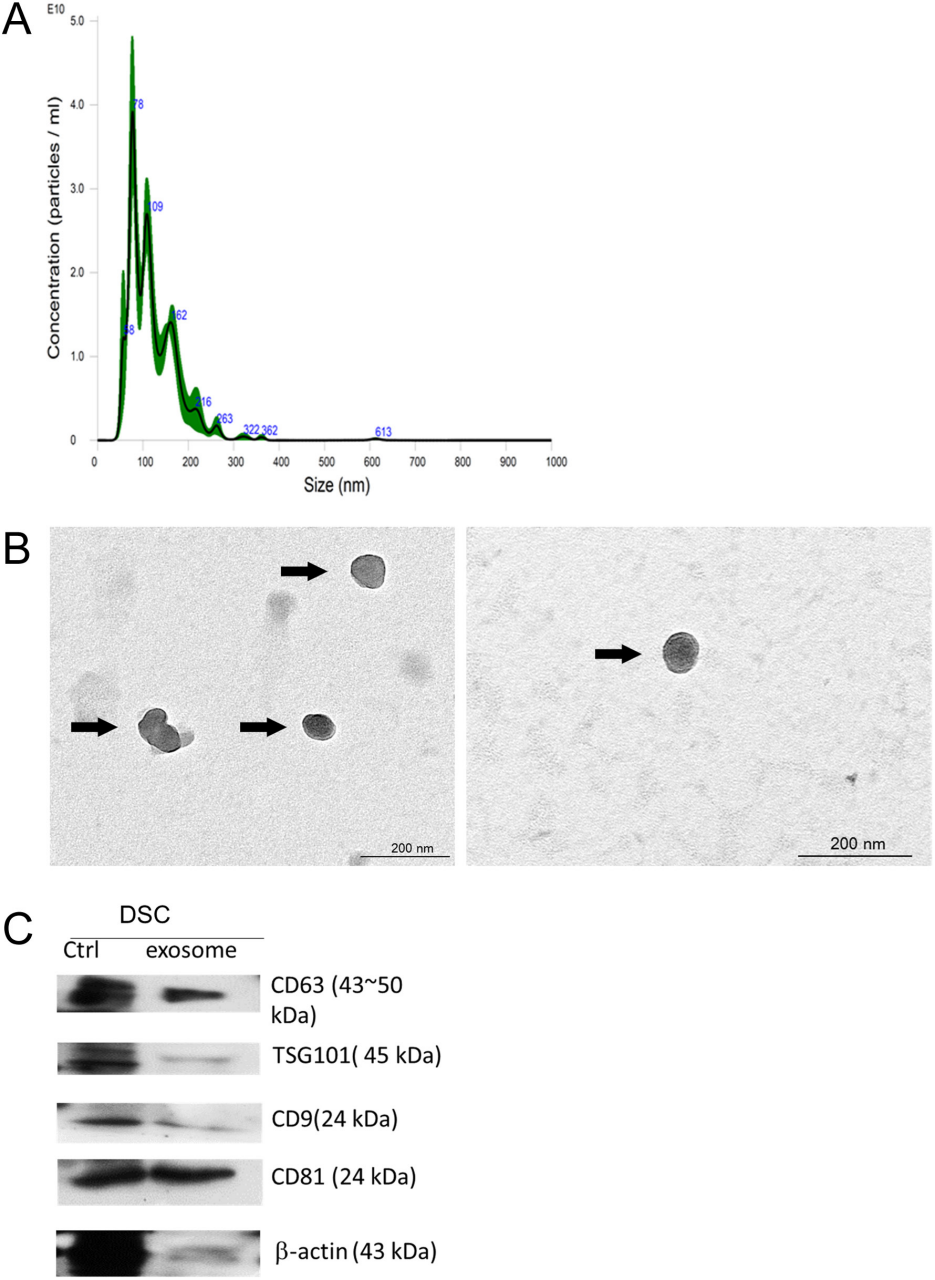
Extracellular vesicles in decidual stromal cells by nanoparticle tracking analysis (NTA), transmission electron microscopy (TEM), and immunoblot analysis. (A) Laser illuminated the nanoparticles, and their movement under Brownian motion was captured for 60 s. The video was subjected to NTA using the NanoSight particle tracking software to calculate nanoparticle concentrations and size distribution. The black line is the average of all data. Green shades are s.d. of three measurements. Blue numbers mean the size (nm) of extracellular vesicles. (B) Isolated extracellular vesicles derived from decidual stromal cells by ultracentrifugation visualized in TEM. In TEM image, EVs (arrow) displayed a round morphology with a lipid bilayer structure. (C) The extracellular vesicle supernatant was denatured in 4× sodium dodecyl sulfate (SDS) buffer and subjected to western blot analysis (10% SDS-PAGE; 50 μg protein/lane) using rabbit CD63, CD9, CD81, and TSG101 antibodies. The control sample contained DSC cell lysate and the exosome sample contained purified exosomes. Beta-actin served as a control. DSC, decidual stromal cells.

By applying nanoparticle tracking analysis (NTA), a newly verified method for the purpose (Witwer *et al.* 2013, Lotvall *et al.* 2014), extracted and purified EVs from decidual stromal cells (DSC) were characterized (Fig. 1A). NTA identified vesicles with a diameter ranging from 40 to 130 nm (Fig. 1A). TEM identified vesicles with a round shape that is characterisitc of EVs (Fig. 1B). Meanwhile, EVs were recognized with positive EV protein markers CD9, TSG101, CD81, and CD63 (Fig. 1C) (Kowal *et al.* 2016).

### Impacts of miRNA-138-5p on GPR124, NLRP3, IL-18, IL-1β inflammasome, and LIF on human decidual stromal cells

**Figure 2:**
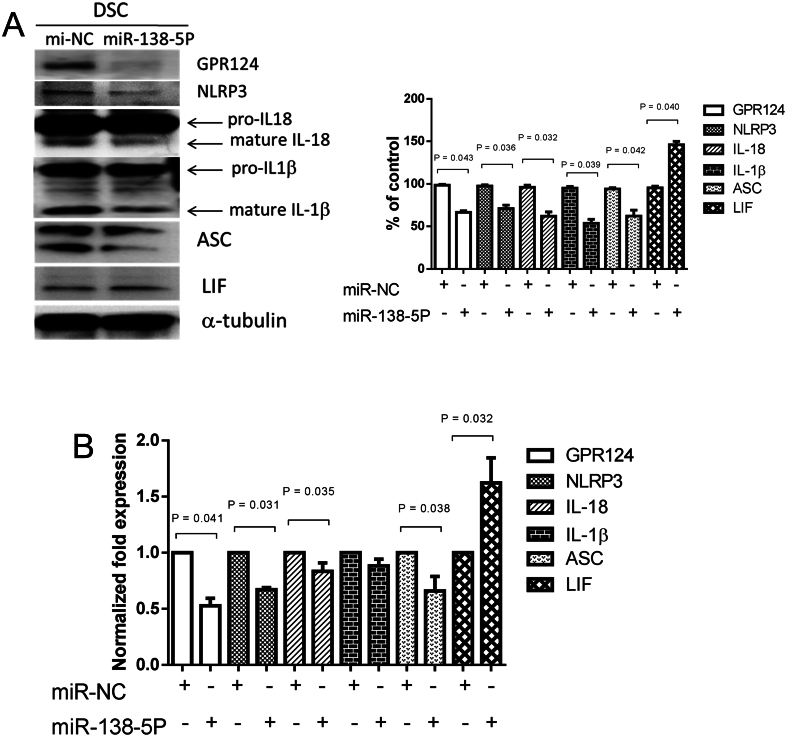
Effects of miRNA-138-5p on GPR124, NLRP3, IL-18, IL-1β inflammasome, and LIF on human decidual stromal cells. Human decidual stromal cells were transfected with miR-138-5P or miR-NC (control) (50 nM) for 48 h. The decidual stromal cells and the culture medium were collected at 48 h after transfection to examine the expression of GPR124, NLRP3, IL-18, IL-1β, and LIF. (A) Immunoblot analysis for protein expression in decidual stromal cells. Expression levels quantified by densitometry. IL-18 and IL-1β in the chart are mature forms that have been quantified. (B) ELISA detection of protein concentration in collected culture medium. All expression results shown as mean ± s.e.m. of three independent experiments, normalized to miR-NC expression, and differences assessed by paired *t*-tests (*P < 0.05 , versus control). DSC, decidual stromal cells.

In our previous report (Wu *et al.* 2022), we applied next-generation sequencing and microarrays to analyze miRNAs. The Gene Expression Omnibus number is GSE203420. Furthermore, we performed the well-established bioinformatics prediction programs TargetScan to identify the target genes and their pathway of the differentially expressed miRNAs. The expression of miR-138-5p and downstream GPR124 in human decidual stromal cells was identified (Wu *et al.* 2022). Based on the findings of our previous study, human decidual stromal cells were transfected with miR-138-5P. After transfection of miR-138-5P, the expression of GPR124, NLRP3, IL-18, IL-1β, and LIF was examined. The protein expressions of GPR124, NLRP3, IL-18, ASC, IL-1β, and LIF were regulated by miR-138-5p in decidual stromal cells as shown by immunoblot analysis (Fig. 2A). IL-18, IL-1β, GPR124, and NLRP3 were downregulated by miR-138-5p; conversely, LIF was upregulated by miR-138-5p. ELISA for collected culture medium was applied to investigate the protein levels of IL-1β, ASC, GPR124, NLRP3, IL-18, and LIF in decidual stromal cells (Fig. 2B). According to the protein levels evaluated by immunoblot and ELISA analyses, the expressions of IL-18, NLRP3, GPR124, and IL-1β were downregulated by miR-138-5p; conversely, LIF was upregulated by miR-138-5p in decidual stromal cells.

### GPR124-regulated expression of IL-18, IL-1β, NLRP3 inflammasome, and LIF in endometrial decidual stromal cells

**Figure 3:**
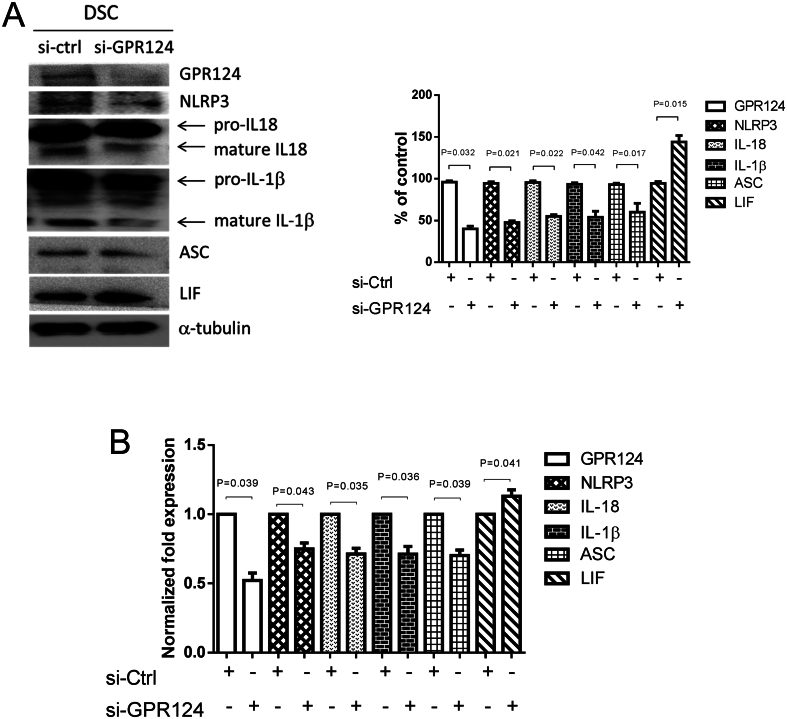
GPR124-regulated expression of NLRP3, IL-18, IL-1β inflammasome, and LIF in endometrial decidual stromal cells. Endometrial decidual stromal cells were transfected with control siRNA (si-ctrl) or GPR124 siRNA (si-GPR124) (50 nM) for 48 h. Decidual stromal cells and the culture medium were collected 48 h after transfection to examine protein expression of GPR124, NLRP3, IL-18, IL-1β, ASC, and LIF. (A) Immunoblot analysis for protein expression in decidual stromal cells. Expression levels quantified by densitometry. IL-18 and IL-1β in the chart are mature forms that have been quantified. (B) ELISA detection of protein concentration in the culture medium. Expression results are shown as mean ± s.e.m. of three independent experiments, normalized to si-ctrl, and differences assessed by paired *t*-tests. DSC, decidual stromal cells.

Endometrial decidual stromal cells were transfected with control siRNA (si-ctrl) and GPR124 siRNA (si-GPR124) for 48 h. The decidual stromal cells and culture medium were collected at 48 h after transfection to examine the expression of IL-18, IL-1β, GPR124, NLRP3, and LIF by immunoblot and ELISA analyses. Pretreatment with GPR124 siRNA abolished the expressions of GPR124, NLRP3, IL-18, and IL-1β in endometrial decidual stromal cells, as shown in Fig. 3A and B. Meanwhile, LIF expression was promoted by GPR124 siRNA pretreatment in decidual stromal cells (Fig. 3A and B).

### NLRP3-regulated expression of IL-18, NLRP3, IL-1β inflammasome, and LIF in endometrial decidual stromal cells

**Figure 4:**
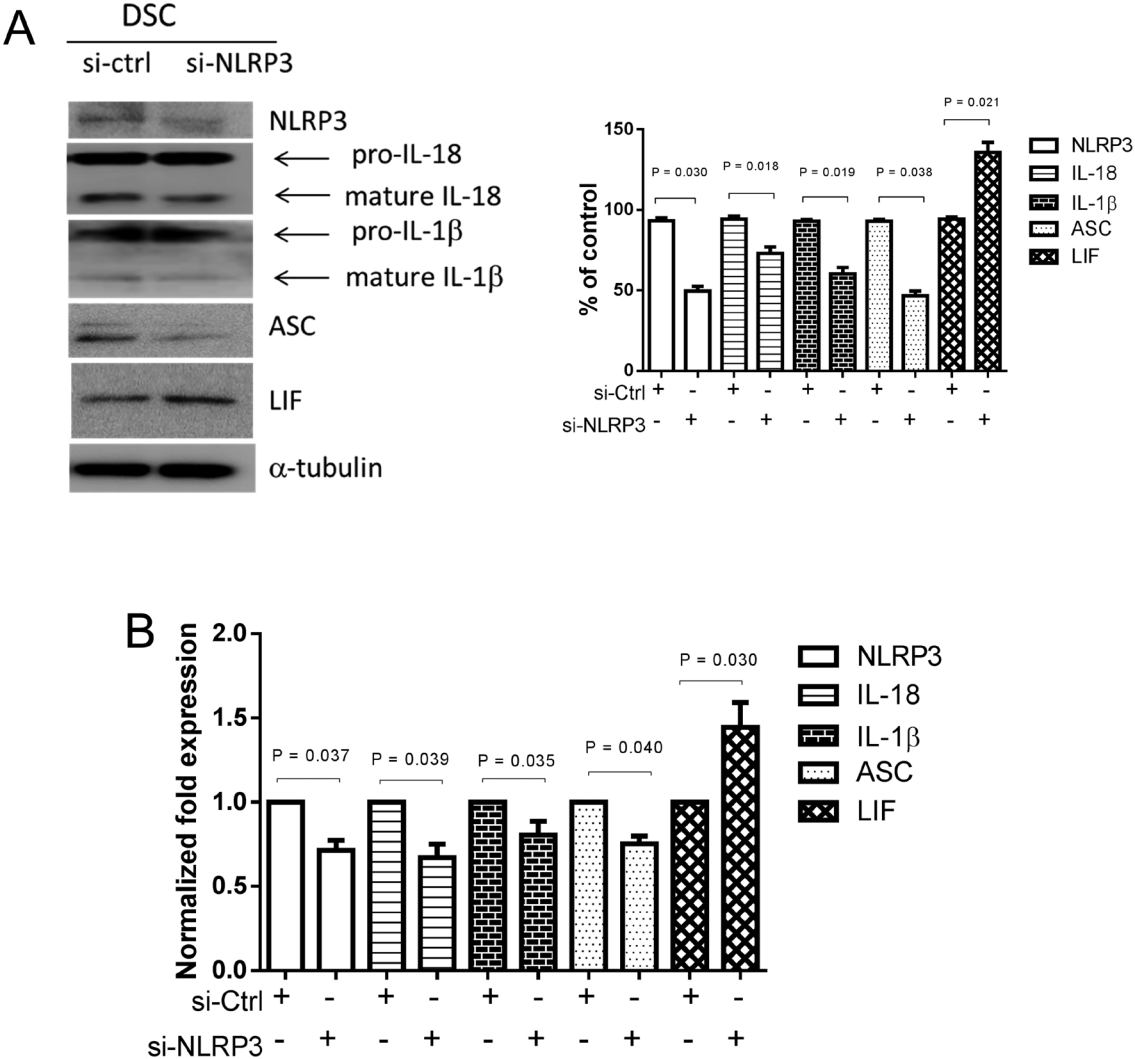
NLRP3-regulated expression of NLRP3, IL-18, IL-1β inflammasome, and LIF in endometrial decidual stromal cells. Endometrial decidual stromal cells were transfected with control siRNA (si-ctrl) and NLRP3 siRNA (si-NLRP3) (50 nM) for 48 h. Decidual stromal cells and the culture medium were collected 48 h after transfection to examine the expression of NLRP3, IL-18, IL-1β, and LIF. (A) Immunoblot analysis for protein expression in decidual stromal cells. Expression levels quantified by densitometry. IL-18 and IL-1β in the chart are mature forms that have been quantified. (B) ELISA detection of protein concentration in the culture medium. Expression results shown as mean ± s.e.m. of three independent experiments, normalized to si-ctrl, and differences assessed by paired *t*-tests. DSC, decidual stromal cells.

Endometrial decidual stromal cells were transfected with control siRNA (si-ctrl) and NLRP3 siRNA (si- NLRP3) for 48 h. The decidual stromal cells and culture medium were collected at 48 h after transfection to examine the expression of IL-18, NLRP3, IL-1β, and LIF by immunoblot and ELISA analyses. Pretreatment with NLRP3 siRNA weakened the expressions of IL-18, NLRP3, and IL-1β in endometrial decidual stromal cells (Fig. 4A and B). Furthermore, the protein expression of LIF was enhanced by NLRP3 siRNA pretreatment in decidual stromal cells (Fig. 4A and B).

### Impacts of miRNA-138-5p on GPR124, LIF, p-STAT3, and STAT3 on human decidual stromal cells

**Figure 5:**
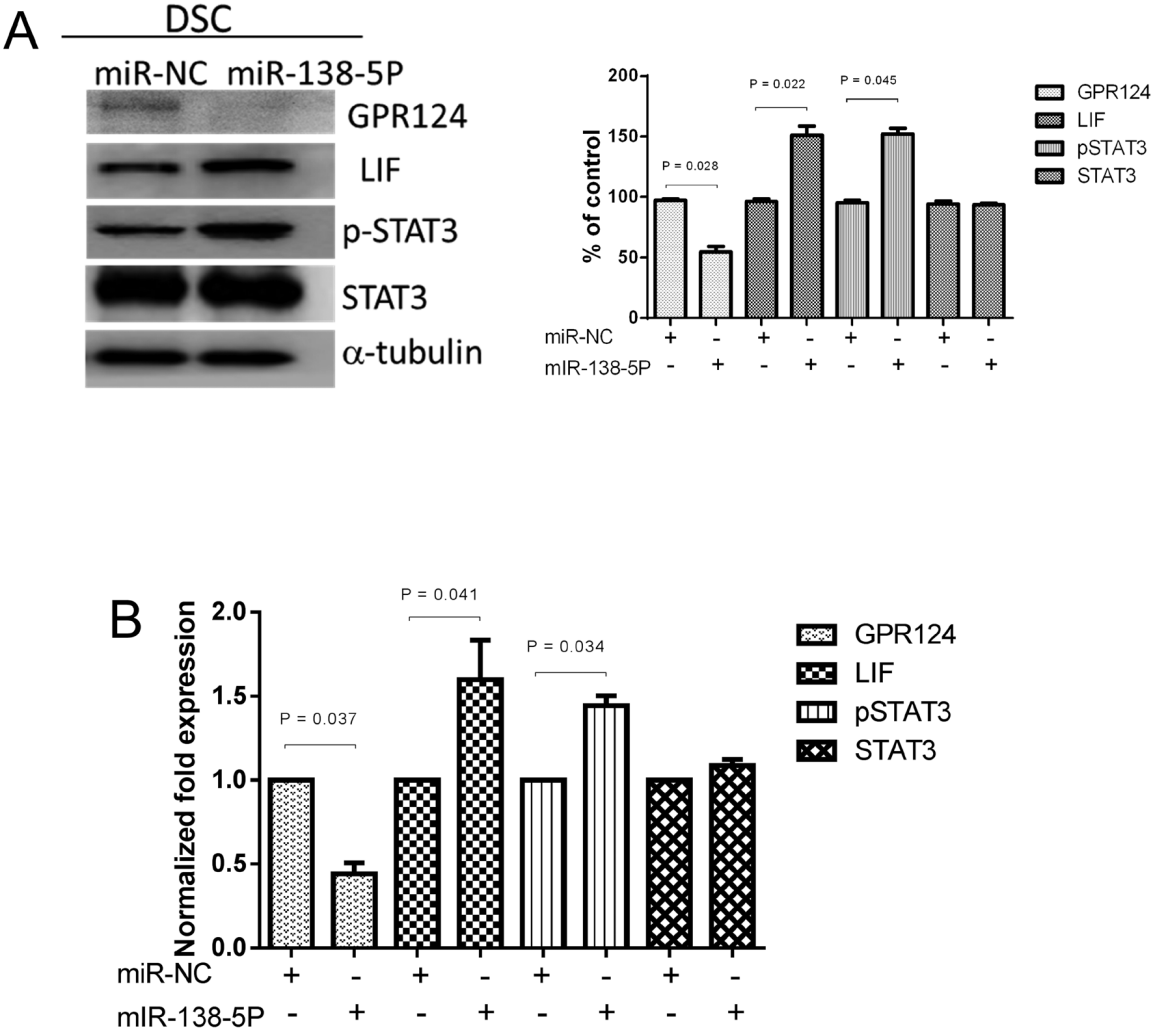
Impacts of miRNA-138-5p on GPR124, LIF, p-STAT3, and STAT3 on human decidual stromal cells. Human decidual stromal cells were transfected with miR-138-5p or miR-NC (50 nM) for 48 h. Decidual stromal cells and the culture medium were collected 48 h after transfection to examine the expression of GPR124, LIF, p-STAT3, and STAT3. (A) Immunoblot analysis for protein expression in decidual stromal cells. Expression levels quantified by densitometry. (B) ELISA detection of protein concentration in culture medium. Expression results shown as mean ± s.e.m. of three independent experiments, normalized to si-ctrl, and differences assessed by paired *t*-tests. DSC, decidual stromal cells.

Human decidual stromal cells were transfected with miR-138-5P (50 nM) for 48 h. The decidual stromal cells and culture medium were collected at 48 h after transfection to examine the expression of GPR124, LIF, p-STAT3, and STAT3 by immunoblot and ELISA analyses. Pretreatment with miR-138-5P weakened the expressions of GPR124 in endometrial decidual stromal cells, as shown in Fig. 5A and B. Moreover, the protein expressions of LIF, p-STAT3, and STAT3 were promoted by miR-138-5P pretreatment in decidual stromal cells, as shown in Fig. 5A and B.

### GPR124-regulated expression of GPR124, LIF, p-STAT3, STAT3 and intergrin β in endometrial decidual stromal cells

**Figure 6:**
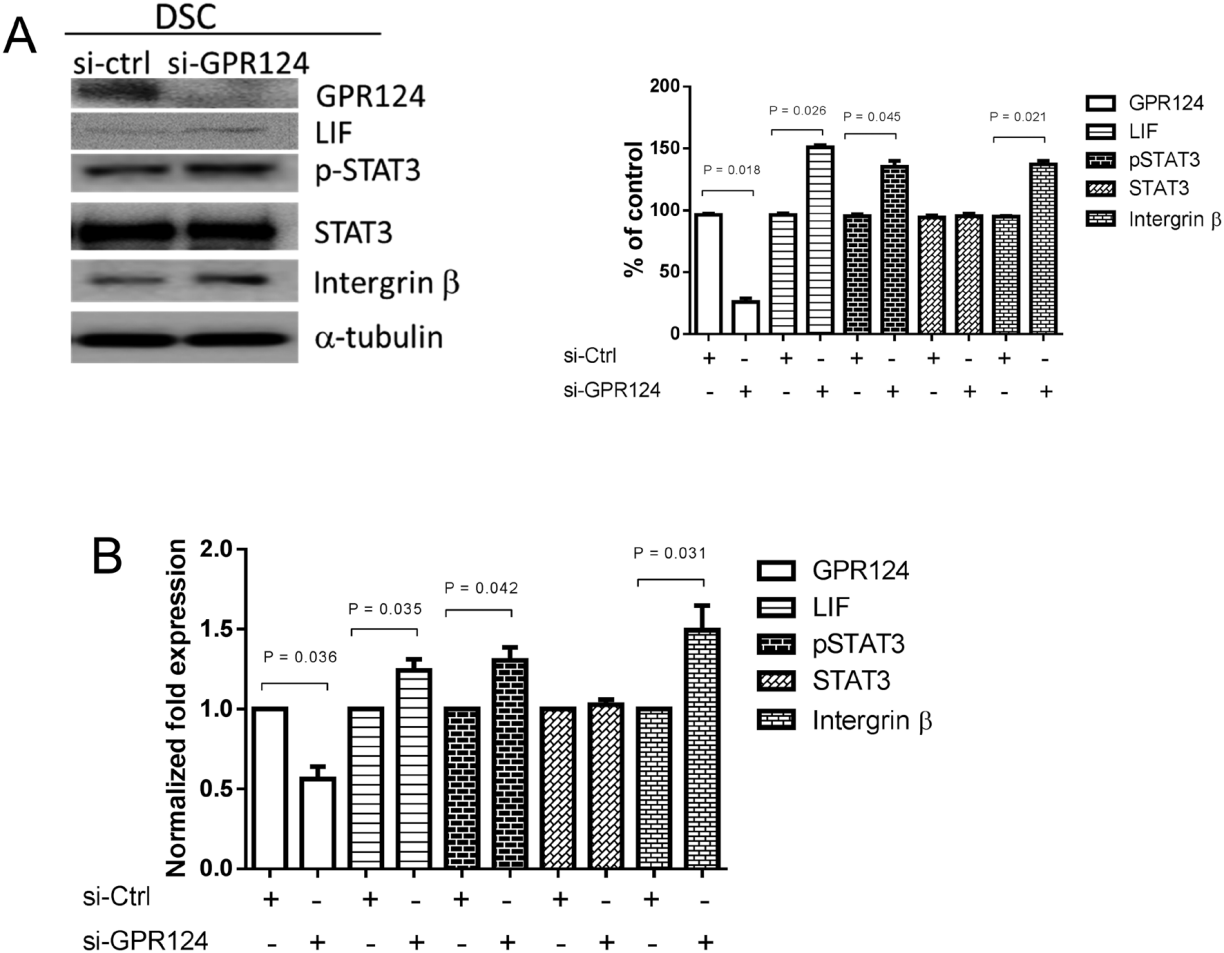
GPR124-regulated expression of GPR124, LIF, p-STAT3, STAT3, and integrin β in endometrial decidual stromal cells. Endometrial decidual stromal cells were transfected with control siRNA (si-ctrl) or GPR124 siRNA (si-GPR124) (50 nM) for 48 h. Decidual stromal cells and the culture medium were collected 48 h after transfection to examine the expression of GPR124, LIF, p-STAT3, STAT3, and integrin β. (A) Immunoblot analysis for protein expression in decidual stromal cells. Expression levels were quantified by densitometry. (B) ELISA detection of protein concentration in culture medium. Expression results shown as mean ± s.e.m. of three independent experiments, normalized to si-ctrl, and differences assessed by paired *t*-tests. DSC, decidual stromal cells.

Endometrial decidual stromal cells were transfected with control siRNA (si-ctrl) and GPR124 siRNA (si-GPR124) for 48 h. The decidual stromal cells and culture medium were collected at 48 h after transfection to examine the expression of GPR124, LIF, p-STAT3, STAT3, and intergrin β by immunoblot and ELISA analyses. Pretreatment with GPR124 siRNA weakened the expressions of GPR124 in endometrial decidual stromal cells, as shown in Fig. 6A and B. Moreover, the protein expressions of LIF, p-STAT3, STAT3, and integrin β were increased by GPR124 siRNA pretreatment in decidual stromal cells, as shown in Fig. 6A and B.

### NLRP3-regulated expression of NLRP3, LIF, p-STAT3, STAT3, and intergrin β in endometrial decidual stromal cells

**Figure 7:**
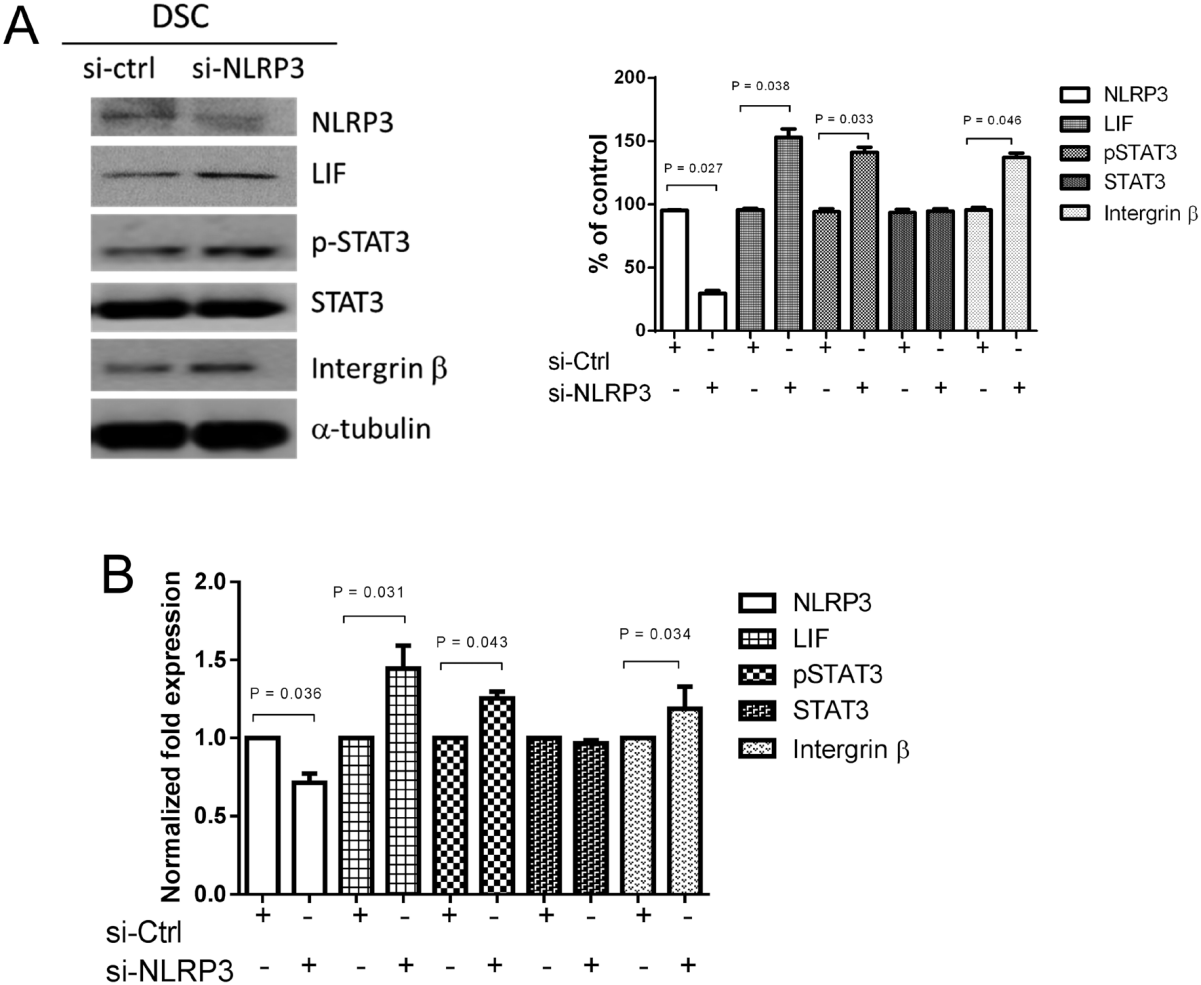
NLRP3-regulated expression of NLRP3, LIF, p-STAT3, STAT3, and integrin β in endometrial decidual stromal cells. Endometrial decidual stromal cells were transfected with control siRNA (si-ctrl) and NLRP3 siRNA (si-NLRP3) (50nM) for 48 h. Decidual stromal cells and the culture medium were collected 48 h after transfection to examine the expression of NLRP3, LIF, p-STAT3, STAT3, and integrin β. (A) Immunoblot analysis for protein expression in decidual stromal cells. Expression levels quantified by densitometry. (B) ELISA detection of protein concentration in culture medium. Expression results shown as mean ± s.e.m. of three independent experiments, normalized to si-ctrl, and differences assessed by paired *t*-tests. DSC, decidual stromal cells.

Endometrial decidual stromal cells were transfected with NLRP3 siRNA (si-NLRP3) and control siRNA (si-ctrl) for 48 h. The decidual stromal cells and culture medium were collected at 48 h after transfection to examine the expression of NLRP3, LIF, p-STAT3, STAT3, and intergrin β by immunoblot and ELISA analyses. Pretreatment with NLRP3 siRNA abolished the expressions of NLRP3 in endometrial decidual stromal cells, as shown in Fig. 7A and B. Meanwhile, the protein expressions of LIF, p-STAT3, STAT3, and integrin β were promoted by NLRP3 siRNA pretreatment in decidual stromal cells, as shown in Fig. 7A and B.

## Discussion

**Figure 8:**
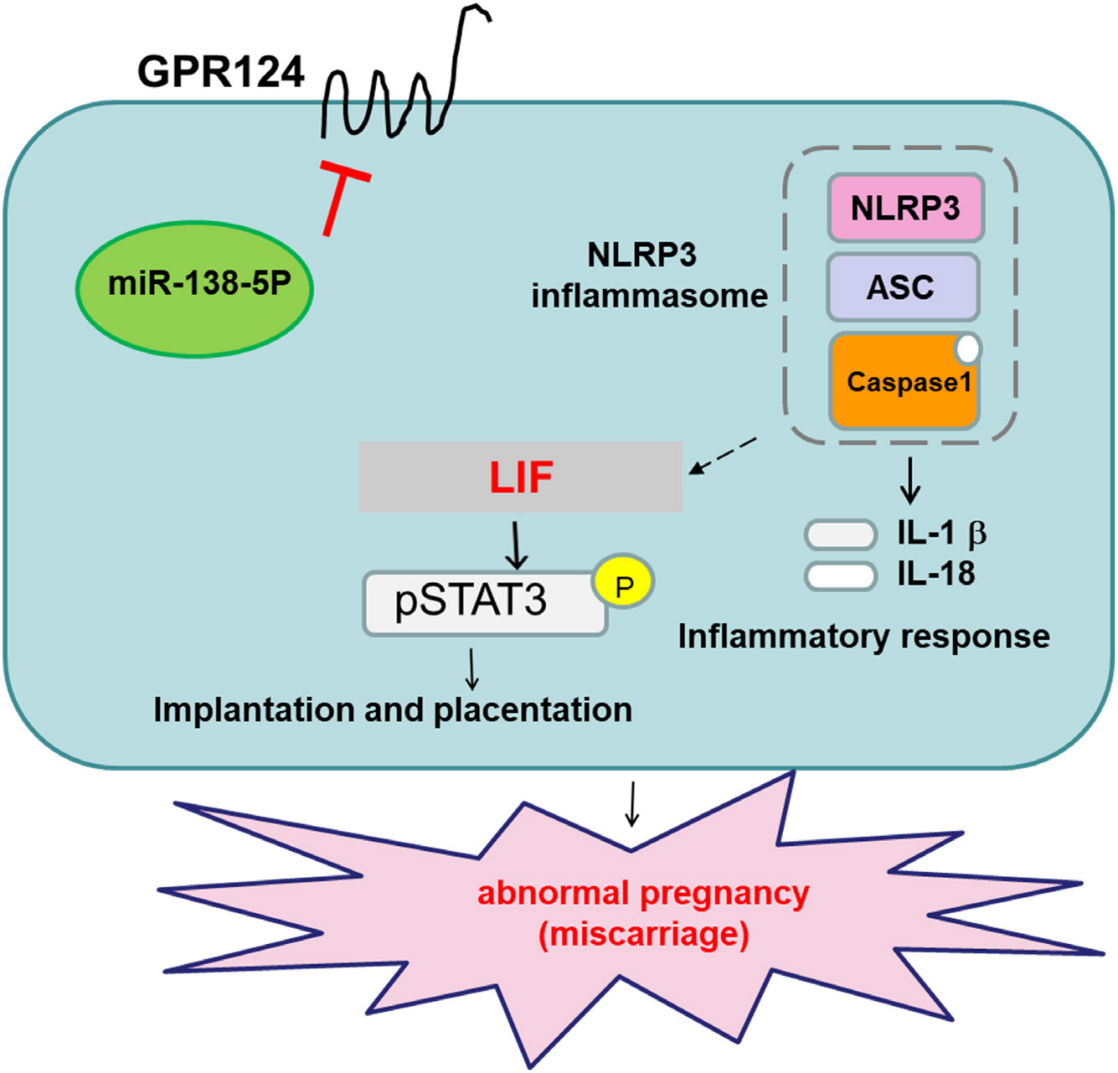
The proposed signaling pathways involved in the leukaemia inhibitory factor–STAT signaling and adhesion molecules in embryo implantation and early pregnancy. These results reveal the vital function of microRNA138-5p and GPR124 in modulating embryo implantation and early pregnancy through the mediation of LIF–STAT signaling and adhesion molecules and the subsequent activation of NLRP3 inflammasome, IL-18, and IL-1β signaling.

Collectively, our findings demonstrated that the purified EVs from decidual stromal cells, the microRNA-138-5p-inhibited GPR124 and inflammasome expression, and microRNA-138-5p activated the expression of LIF–STAT and adhesion molecules in human decidual stromal cells. Additionally, the knockdown of GPR124 and NLRP3 through siRNA increases the expression of LIF–STAT and adhesion molecules (Fig. 8). The embryo–maternal cross talk during embryo implantation and early pregnancy plays an essential role in the control of human reproduction. Many protocols have been created to induce decidualization; however, the period of challenge varies markedly between studies, ranging from a couple of days to 10 or more days (Gellersen & Brosens 2014). The heterogeneity in experimental design with the diverse extent of cellular reprogramming upon decidualization (Gellersen & Brosens 2003) is the rationale for designing this study to be conducted using human decidual stromal cells collected from the decidual tissue. EVs and cargo of EVs between cells have been considered critical factors for embryo implantation and programming of human pregnancy. This study aimed to evaluate the functional embryo–maternal cross talk during the embryo implantation and placentation through EV-carried microRNA-138-5p, downstream GPR124-regulated inflammasome, LIF–STAT, and adhesion molecules signaling in human decidual stromal cells. LIF is a member of the IL-6 cytokine family. The biological role of the LIF in reproduction has been characterized previously, including blastocyst development, initiating embryo–uterine communication, implantation, and trophoblast invasion (Nicola & Babon 2015). The expression of LIF was demonstrated in the endometrium in fertile and infertile women, while a differential expression of LIF was observed in women with implantation failure and infertility (Wu *et al.* 2013, Zare *et al.* 2020). EVs are involved in the complex biological processes of cell–cell interactions, including embryo–endometrial interactions. The present evidence demonstrates that EVs integrate into the target cell’s plasma membrane and secrete their cargo, such as miRNAs, into the cell, where they can instantly interact with the target cell (Thery *et al.* 2002, Morelli *et al.* 2004). Our findings showed that extracted EVs from decidua and decidual stromal cells were investigated by immunoblot analysis, TEM, and nanoparticle tracking analysis. These EVs may contain miRNAs that can contribute to embryo implantation and pregnancy. miRNAs, a flock of non-coding RNAs, play a role in controlling the expression of target genes by partly or wholly binding to their independent complementary sequences (Zammar *et al.* 2014, Araldi & Suarez 2016). The expression profiles of miRNAs in embryo implantation and early pregnancy may have a role in some pathological regulation, possibly specific to spontaneous miscarriage. The impacts of some miRNAs in specific actions concerning the pathophysiology of spontaneous miscarriage have been shown in several studies with different mechanisms, such as reducing the proliferation and migration of endothelial cells and smooth muscle cells (Ferreira *et al.* 2014, Zammar *et al.* 2014, Huang *et al.* 2017). The contribution of miRNAs in regulating underlying mechanisms associated with abnormal embryo implantation and pregnancy needs to be well investigated. In our previous study (Wu *et al.* 2022), we demonstrated differential expression of miRNAs between normal pregnancy and spontaneous miscarriage in a hierarchical clustering analysis by microarray analysis of miRNAs in the endometrial tissue, where we showed that miRNA-138-5p is downregulated in spontaneous miscarriage compared to normal pregnancy. MicroRNA-138-5p not only has a vital role in the brain but also in peripheral tissues (Zottel *et al.* 2020). Furthermore, gene ontology analysis showed that microRNA-138-5p promotes physiological actions such as angiogenesis, cell migration, and placenta establishment, suggesting that the malfunction in these physiological actions contributes to abnormal embryo implantation and pregnancy (Lala & Nandi 2016, Salomon *et al.* 2017). In our previous study (Wu *et al.* 2022), we searched the target genes of miR-138-5p by using established miRNA target prediction programs and identified GPR124 as a potential target of miR-138-5p using TargetScan. GPR124 is an orphan G protein-coupled receptor that functionally modulates physiological angiogenesis and embryonic alteration within the central nervous system (Erkinova *et al.* 2018, Florian *et al.* 2020). The physiological role of GPR124 is established in angiogenesis and atherosclerosis potentially associated with embryo implantation. Our previous results demonstrated an opposite interaction between the expression of miR-138-5p and GPR124 in endometrial tissues (Wu *et al.* 2022). Our previous results propose that the interaction of miR-138-5p and GPR124 results in embryo implantation and early pregnancy; however, the underlying mechanism of this interaction still needs to be investigated.

Inflammasomes trigger inflammatory actions, which subsequently activate pro-inflammatory cytokines IL-1β and IL-18, and intercede cytokine production and programmed cell fate (Liu *et al.* 2016, Shi *et al.* 2017). Here, we showed the increased expression of NLRP3 inflammasome, IL-18, and IL-1β in spontaneous miscarriage compared to normal pregnancy; meanwhile, the expressions of IL-18, NLRP3, and IL-1β are modulated by miR138-5p and GRP124 in decidual stromal cells, indicating that IL-18 inflammasome, NLRP3, and IL-1β could be related to abnormal pregnancy. Furthermore, in the present study, we demonstrate for the first time that miR138-5p and GRP124 may participate in early pregnancy by mediating the expression of NLRP3 inflammasome, IL-18, and IL-1β, exploring the complex process of embryo implantation and early pregnancy.

Based on the evidence of this study, the challenge of human decidual stromal cells with microRNA138-5p leads to significantly increased activation of LIF, p-STAT3, STAT3, and integrin β; meanwhile, reduced expression of NLRP3, GPR124, IL-1β, and IL-18. Our results indicate that miRNA138-5p directly controls the expression of LIF, p-STAT3, STAT3, NLRP3, integrin β, GPR124, IL-1β, and IL-18 in human decidual stromal cells. At the same time, in this study, GPR124 siRNA was applied to specifically knock down the protein expression of GPR124 in human decidual stromal cells. Contrary to GPR124 with siRNA weakened the GPR124-modulated activation of NLRP3, IL-18, IL-1β, LIF, p-STAT3, STAT3, and integrin β in human decidual stromal cells, suggesting that the impacts of GPR124 on embryo implantation and early pregnancy are through the expression of NLRP3, IL-18, IL-1β, LIF, p-STAT3, STAT3, and integrin β. Meanwhile, we also used NLRP3 siRNA to selectively knock down the protein expression of NLRP3 in human decidual stromal cells. NLRP3 siRNA reduced the NLRP3-promoted activation of IL-18, IL-1β, and reversed the NLRP3-inhibited activation of LIF, p-STAT3, STAT3, and integrin β in human decidual stromal cells. Taken together, exploring the actions of microRNA138-5p by manipulating GPR124 and downstream inflammasome and LIF–STAT3 signaling in decidual stromal cells may clarify the complex process of embryo implantation and early pregnancy, which is one of the novel findings in this study.

In summary, in this study, we have identified the regulation of miRNA138-5p in embryo implantation and early pregnancy by modifying GPR124 expression, inflammasome signaling, and downstream LIF–STAT3 signaling (Fig. 8). These results imply that the miRNA138-5p-modulating GPR124, NLRP3 inflammasome, and LIF–STAT3 signaling in the decidual endometrium constitute a potential therapeutic approach for enhancing embryo implantation in the treatment of infertility but also would provide important insights into the mechanism of embryo implantation and decidual programming of human pregnancy.

## Declaration of interest

The authors declare that they have no competing interests.

## Funding

This work was supported by grants MOST-106-2314-B-182A-166-, MOST-107-2314-B-182A-098-, and MOST-109-2314-B-182A-096- (to H-MW) from the Ministry of Science and Technologyhttp://dx.doi.org/10.13039/501100003711, Taiwan, and grants CMRPG310421 and CMRPG3J1761 (to H-MW).

## Ethics approval and consent to participate

This study was approved by the Institutional Review Board of Chang Gung Memorial Hospital (CGMH-IRB numbers 201601676A3, 201702112B0, 201802242A3, 201902015B0, 202002376B0, and 202100385B0).

## Data availability

The datasets used and analyzed during the current study are available from the corresponding author on reasonable request.

## Consent for publication

All authors consent to the publication of this article.

## Author contribution statement

H-MW performed the experiments, interpreted the results and prepared the manuscript. H-MW, LHC, WJC, and CLT contributed to scientific discussion and manuscript editing. HMW and CLT supervised in the design of the study and finalized the manuscript. All authors read and approved the final version of the manuscript.

## Acknowledgements

The authors acknowledge WJ Qiu and FW Chen at the Gynecological and Obstetrics Laboratory, Chang Gung Memorial Hospital, for their technical assistance in this study.

## References

[bib1] Araldi E & Suarez Y 2016 MicroRNAs as regulators of endothelial cell functions in cardiometabolic diseases. Biochimica et Biophysica Acta 1861 2094–2103. (10.1016/j.bbalip.2016.01.013) 26825686 PMC5039046

[bib2] Blois SM Kammerer U Alba Soto C Tometten MC Shaikly V Barrientos G Jurd R Rukavina D Thomson AW Klapp BF, *et al.* 2007 Dendritic cells: key to fetal tolerance? Biology of Reproduction 77 590–598. (10.1095/biolreprod.107.060632) 17596562

[bib3] Ceydeli N Kaleli S Calay Z Erel CT Akbas F & Ertungealp E 2006 Difference in alpha(v)beta3 integrin expression in endometrial stromal cell in subgroups of women with unexplained infertility. European Journal of Obstetrics and Gynecology and Reproductive Biology 126 206–211. (10.1016/j.ejogrb.2005.11.034) 16386348

[bib4] Chou CS MacCalman CD & Leung PC 2003 Differential effects of gonadotropin-releasing hormone I and II on the urokinase-type plasminogen activator/plasminogen activator inhibitor system in human decidual stromal cells in vitro. Journal of Clinical Endocrinology and Metabolism 88 3806–3815. (10.1210/jc.2002-021955) 12915673

[bib5] Cullinan EB Abbondanzo SJ Anderson PS Pollard JW Lessey BA & Stewart CL 1996 Leukemia inhibitory factor (LIF) and LIF receptor expression in human endometrium suggests a potential autocrine/paracrine function in regulating embryo implantation. PNAS 93 3115–3120. (10.1073/pnas.93.7.3115) 8610178 PMC39771

[bib6] Erkinova SA Sokolova EA Orlov KY Kiselev VS Strelnikov NV Dubovoy AV Voronina EN & Filipenko ML 2018 Angiopoietin-like proteins 4 (ANGPTL4) gene polymorphisms and risk of brain arteriovenous malformation. Journal of Stroke and Cerebrovascular Diseases 27 908–913. (10.1016/j.jstrokecerebrovasdis.2017.10.033) 29221972

[bib7] Ferreira R Santos T Amar A Tahara SM Chen TC Giannotta SL & Hofman FM 2014 MicroRNA-18a improves human cerebral arteriovenous malformation endothelial cell function. Stroke 45 293–297. (10.1161/STROKEAHA.113.003578) 24203843

[bib8] Fitzgerald JS Poehlmann TG Schleussner E & Markert UR 2008 Trophoblast invasion: the role of intracellular cytokine signalling via signal transducer and activator of transcription 3 (STAT3). Human Reproduction Update 14 335–344. (10.1093/humupd/dmn010) 18424427

[bib9] Florian IA Timis TL Ungureanu G Florian IS Balasa A & Berindan-Neagoe I 2020 Deciphering the vascular labyrinth: role of microRNAs and candidate gene SNPs in brain AVM development - literature review. Neurological Research 42 1043–1054. (10.1080/01616412.2020.1796380) 32723034

[bib10] Gellersen B & Brosens J 2003 Cyclic AMP and progesterone receptor cross-talk in human endometrium: a decidualizing affair. Journal of Endocrinology 178 357–372. (10.1677/joe.0.1780357) 12967329

[bib11] Gellersen B & Brosens JJ 2014 Cyclic decidualization of the human endometrium in reproductive health and failure. Endocrine Reviews 35 851–905. (10.1210/er.2014-1045) 25141152

[bib12] Gellersen B Reimann K Samalecos A Aupers S & Bamberger AM 2010 Invasiveness of human endometrial stromal cells is promoted by decidualization and by trophoblast-derived signals. Human Reproduction 25 862–873. (10.1093/humrep/dep468) 20118488

[bib13] Greening DW Nguyen HP Evans J Simpson RJ & Salamonsen LA 2016 Modulating the endometrial epithelial proteome and secretome in preparation for pregnancy: the role of ovarian steroid and pregnancy hormones. Journal of Proteomics 144 99–112. (10.1016/j.jprot.2016.05.026) 27262222

[bib14] Huang J Song J Qu M Wang Y An Q Song Y Yan W Wang B Wang X Zhang S, *et al.* 2017 MicroRNA-137 and microRNA-195* inhibit vasculogenesis in brain arteriovenous malformations. Annals of Neurology 82 371–384. (10.1002/ana.25015) 28802071

[bib15] Kowal J Arras G Colombo M Jouve M Morath JP Primdal-Bengtson B Dingli F Loew D Tkach M & Thery C 2016 Proteomic comparison defines novel markers to characterize heterogeneous populations of extracellular vesicle subtypes. PNAS 113 E968–977. (10.1073/pnas.1521230113) 26858453 PMC4776515

[bib16] Lala PK & Nandi P 2016 Mechanisms of trophoblast migration, endometrial angiogenesis in preeclampsia: the role of decorin. Cell Adhesion and Migration 10 111–125. (10.1080/19336918.2015.1106669) 26745663 PMC4853052

[bib17] Liu X Zhang Z Ruan J Pan Y Magupalli VG Wu H & Lieberman J 2016 Inflammasome-activated gasdermin D causes pyroptosis by forming membrane pores. Nature 535 153–158. (10.1038/nature18629) 27383986 PMC5539988

[bib18] Lotvall J Hill AF Hochberg F Buzas EI Di Vizio D Gardiner C Gho YS Kurochkin IV Mathivanan S Quesenberry P, *et al.* 2014 Minimal experimental requirements for definition of extracellular vesicles and their functions: a position statement from the International Society for Extracellular Vesicles. Journal of Extracellular Vesicles 3 26913. (10.3402/jev.v3.26913) 25536934 PMC4275645

[bib19] Morelli AE Larregina AT Shufesky WJ Sullivan ML Stolz DB Papworth GD Zahorchak AF Logar AJ Wang Z Watkins SC, *et al.* 2004 Endocytosis, intracellular sorting, and processing of exosomes by dendritic cells. Blood 104 3257–3266. (10.1182/blood-2004-03-0824) 15284116

[bib20] Nicola NA & Babon JJ 2015 Leukemia inhibitory factor (LIF). Cytokine and Growth Factor Reviews 26 533–544. (10.1016/j.cytogfr.2015.07.001) 26187859 PMC4581962

[bib21] Salomon C Guanzon D Scholz-Romero K Longo S Correa P Illanes SE & Rice GE 2017 Placental exosomes as early biomarker of preeclampsia: potential role of exosomal microRNAs across gestation. Journal of Clinical Endocrinology and Metabolism 102 3182–3194. (10.1210/jc.2017-00672) 28531338

[bib22] Shi J Gao W & Shao F 2017 Pyroptosis: gasdermin-mediated programmed necrotic cell death. Trends in Biochemical Sciences 42 245–254. (10.1016/j.tibs.2016.10.004) 27932073

[bib23] Suman P Shembekar N & Gupta SK 2013 Leukemia inhibitory factor increases the invasiveness of trophoblastic cells through integrated increase in the expression of adhesion molecules and pappalysin 1 with a concomitant decrease in the expression of tissue inhibitor of matrix metalloproteinases. Fertility and Sterility 99 533–542. (10.1016/j.fertnstert.2012.10.004) 23122949

[bib24] Teklenburg G Salker M Molokhia M Lavery S Trew G Aojanepong T Mardon HJ Lokugamage AU Rai R Landles C, *et al.* 2010 Natural selection of human embryos: decidualizing endometrial stromal cells serve as sensors of embryo quality upon implantation. PLoS One 5 e10258. (10.1371/journal.pone.0010258) 20422011 PMC2858159

[bib25] Thery C Zitvogel L & Amigorena S 2002 Exosomes: composition, biogenesis and function. Nature Reviews 2 569–579. (10.1038/nri855) 12154376

[bib26] Witwer KW Buzas EI Bemis LT Bora A Lasser C Lotvall J Nolte-’t Hoen EN Piper MG Sivaraman S Skog J, *et al.* 2013 Standardization of sample collection, isolation and analysis methods in extracellular vesicle research. Journal of Extracellular Vesicles 2 e20360. (10.3402/jev.v2i0.20360) PMC3760646 24009894

[bib27] Wu HM Huang HY Lee CL Soong YK Leung PC & Wang HS 2015 Gonadotropin-releasing hormone type II (GnRH-II) agonist regulates the motility of human decidual endometrial stromal cells: possible effect on embryo implantation and pregnancy. Biology of Reproduction 92 98. (10.1095/biolreprod.114.127324) 25761596

[bib28] Wu HM Lo TC Tsai CL Chen LH Huang HY Wang HS & Yu J 2022 Extracellular vesicle-associated MicroRNA-138-5p regulates embryo implantation and early pregnancy by adjusting GPR124. Pharmaceutics 14 1172. (10.3390/pharmaceutics14061172) 35745744 PMC9230557

[bib29] Wu M Yin Y Zhao M Hu L & Chen Q 2013 The low expression of leukemia inhibitory factor in endometrium: possible relevant to unexplained infertility with multiple implantation failures. Cytokine 62 334–339. (10.1016/j.cyto.2013.03.002) 23541977

[bib30] Zammar SG El Tecle NE El Ahmadieh TY McClendon J Comair YG & Bendok BR 2014 A biological approach to treating brain arteriovenous malformations. Neurosurgery 74 N15–N17. (10.1227/01.neu.0000445336.35080.60) 24642990

[bib31] Zare F Amiri MM Hadinedoushan H Dehghan-Manshadi M Mansouri F Fesahat F & Saboor-Yaraghi AA 2020 Contraceptive and molecular function of a novel recombinant vaccine based human leukemia inhibitory factor on BALB/c mice: an experimental in vivo study. Journal of Reproductive Immunology 142 103195. (10.1016/j.jri.2020.103195) 32927320

[bib32] Zottel A Samec N Kump A Raspor Dall’Olio LR Puzar Dominkus P Romih R Hudoklin S Mlakar J Nikitin D Sorokin M, *et al.* 2020 Analysis of miR-9-5p, miR-124-3p, miR-21-5p, miR-138-5p, and miR-1-3p in glioblastoma cell lines and extracellular vesicles. International Journal of Molecular Sciences 21 8491. (10.3390/ijms21228491) 33187334 PMC7698225

